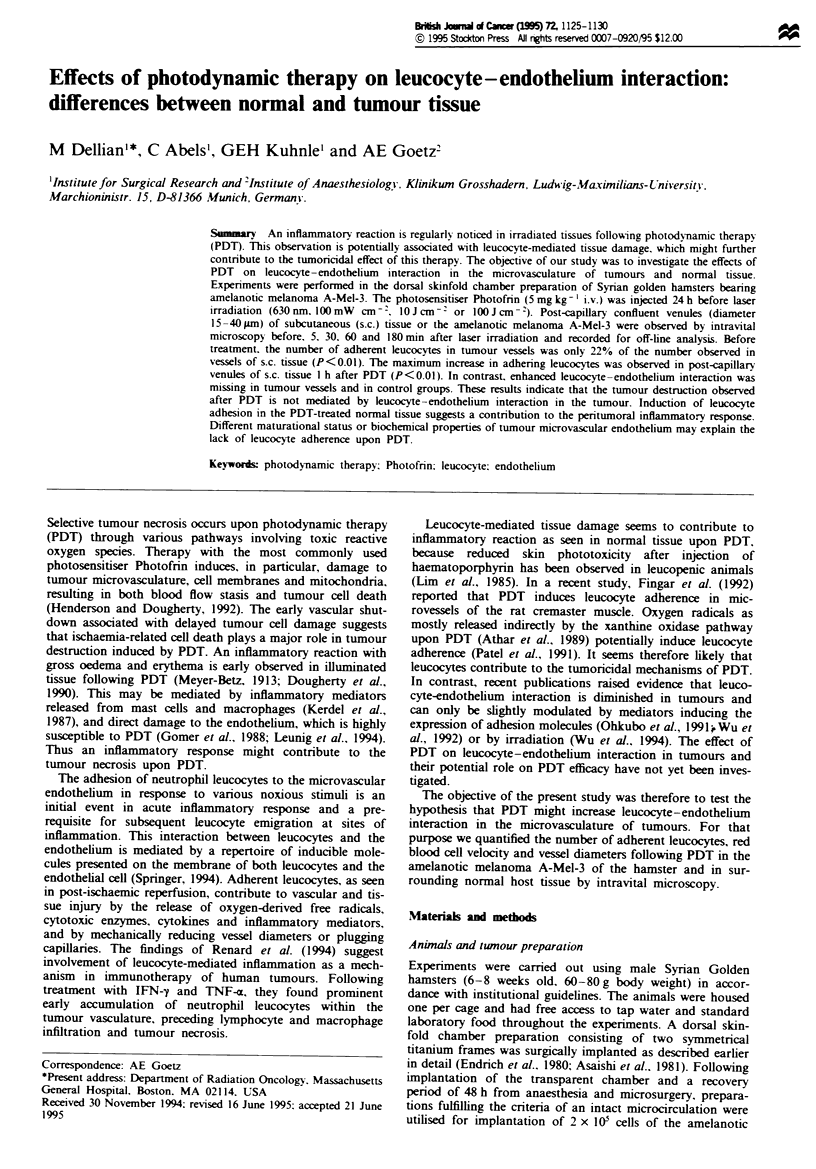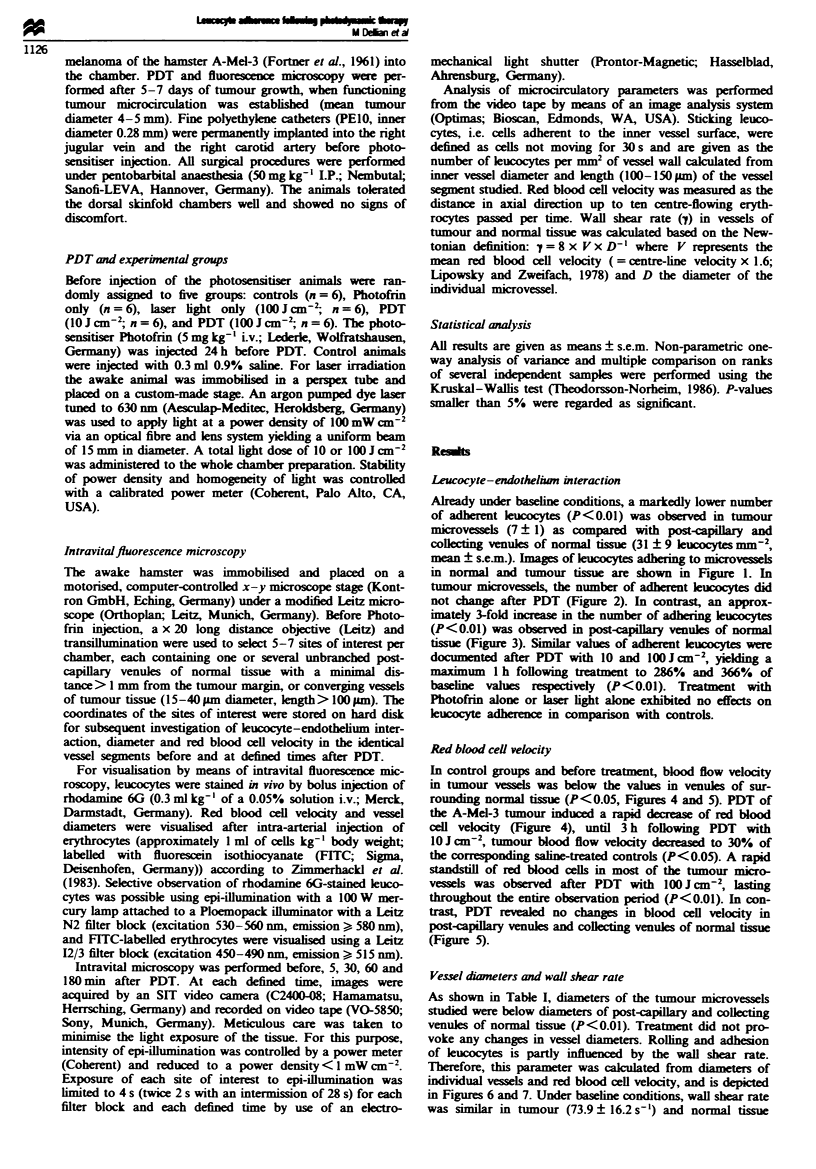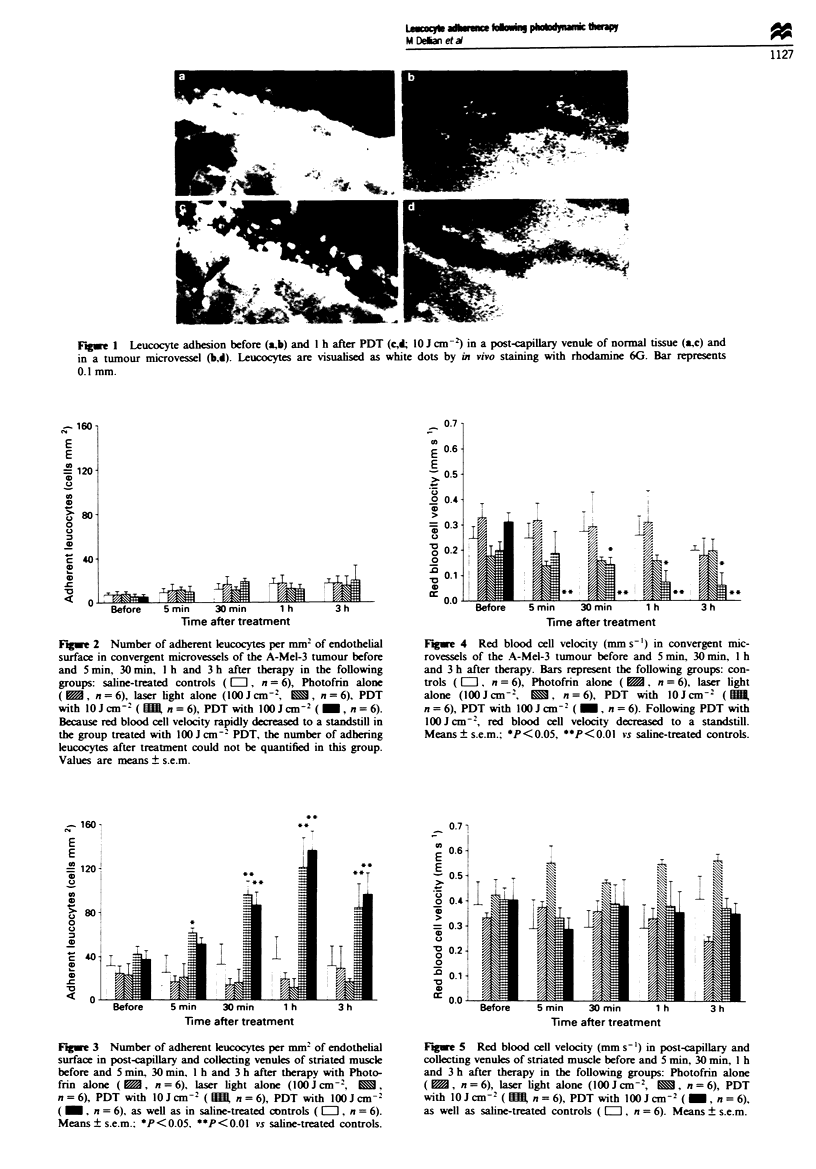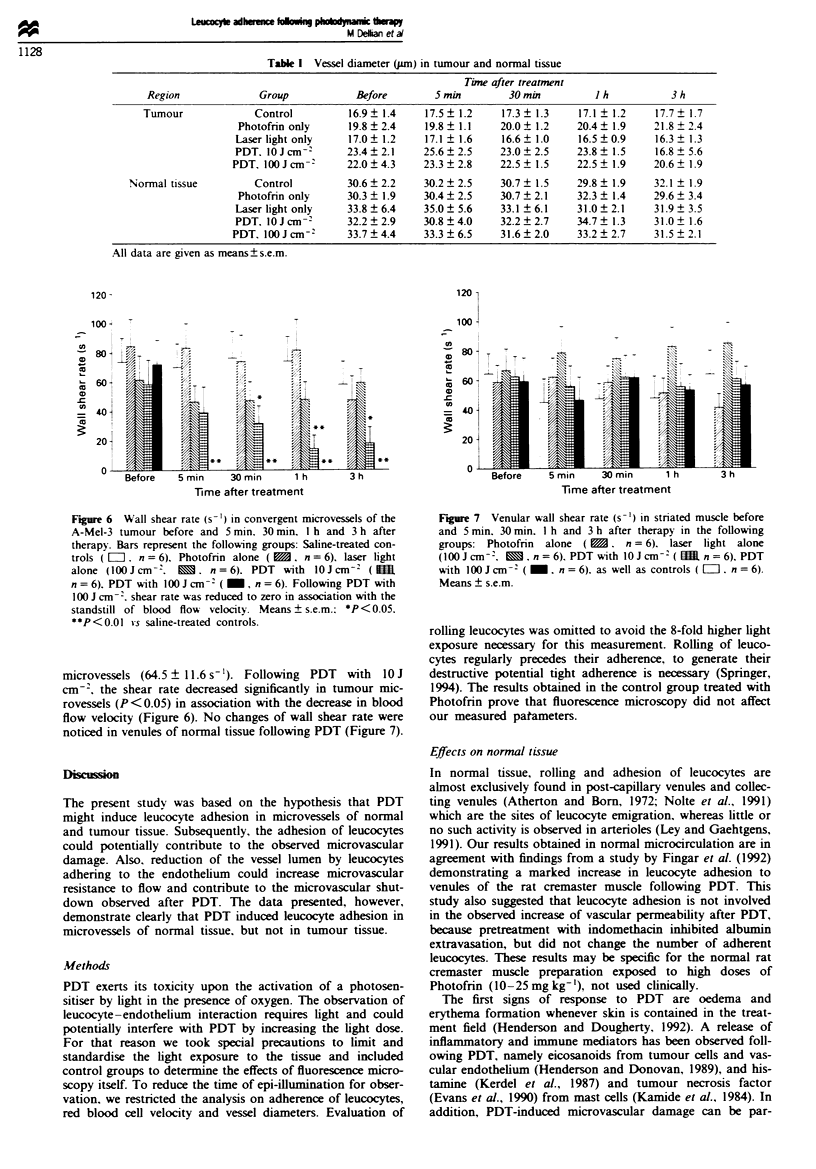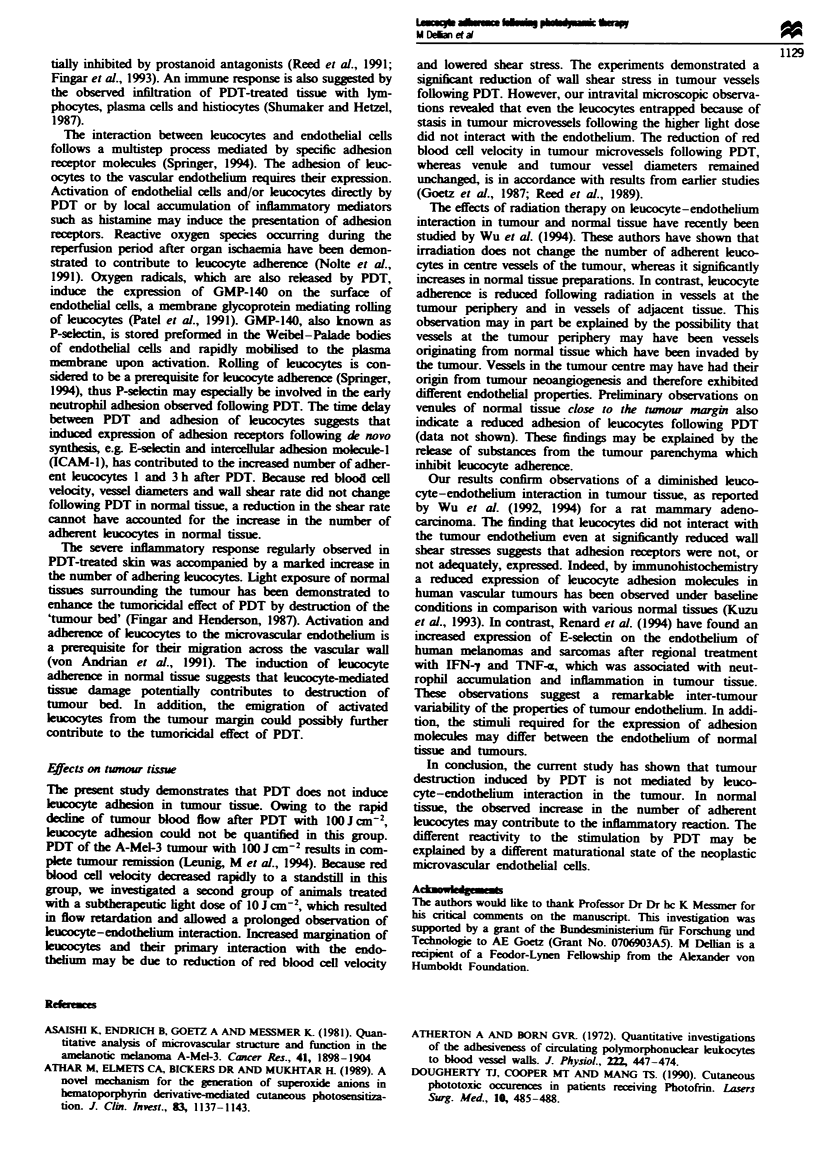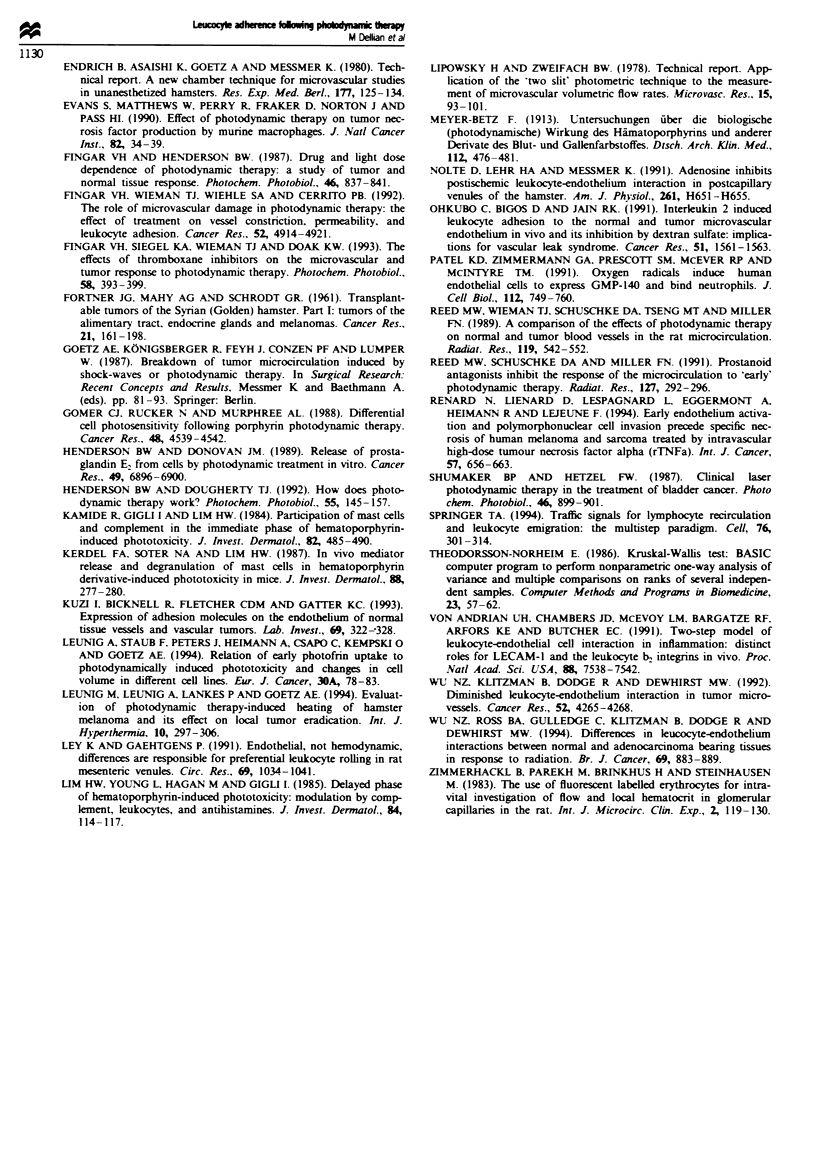# Effects of photodynamic therapy on leucocyte-endothelium interaction: differences between normal and tumour tissue.

**DOI:** 10.1038/bjc.1995.475

**Published:** 1995-11

**Authors:** M. Dellian, C. Abels, G. E. Kuhnle, A. E. Goetz

**Affiliations:** Institute for Surgical Reserch, Klinikum Grosshadern, Ludwig-Maximilians-University, Munich, Germany.

## Abstract

**Images:**


British Journal of Cancer (1995) 72, 1125-1130

© 1995 Stockton Press All rights reserved 0007-0920/95 $12.00

### Effects of photodynamic therapy on leucocyte–endothelium interaction: differences between normal and tumour tissue

M Dellian[1]*, C Abels[1], GEH Kuhnle[1] and AE Goetz[2]

[1]Institute for Surgical Research and [2]Institute of Anaesthesiology, Klinikum Grosshadern, Ludwig-Maximilians-University, Marchioninistr. 15, D-81366 Munich, Germany.

**Summary** An inflammatory reaction is regularly noticed in irradiated tissues following photodynamic therapy (PDT). This observation is potentially associated with leucocyte-mediated tissue damage, which might further contribute to the tumoricidal effect of this therapy. The objective of our study was to investigate the effects of PDT on leucocyte–endothelium interaction in the microvasculature of tumours and normal tissue. Experiments were performed in the dorsal skinfold chamber preparation of Syrian golden hamsters bearing amelanotic melanoma A-Mel-3. The photosensitiser Photofrin (5 mg $kg^{-1}$ i.v.) was injected 24 h before laser irradiation (630 nm, 100 mW $cm^{-2}$, 10 J $cm^{-2}$ or 100 J $cm^{-2}$). Post-capillary confluent venules (diameter 15–40 μm) of subcutaneous (s.c.) tissue or the amelanotic melanoma A-Mel-3 were observed by intravital microscopy before, 5, 30, 60 and 180 min after laser irradiation and recorded for off-line analysis. Before treatment, the number of adherent leucocytes in tumour vessels was only 22% of the number observed in vessels of s.c. tissue ($P<0.01$). The maximum increase in adhering leucocytes was observed in post-capillary venules of s.c. tissue 1 h after PDT ($P<0.01$). In contrast, enhanced leucocyte–endothelium interaction was missing in tumour vessels and in control groups. These results indicate that the tumour destruction observed after PDT is not mediated by leucocyte–endothelium interaction in the tumour. Induction of leucocyte adhesion in the PDT-treated normal tissue suggests a contribution to the peritumoral inflammatory response. Different maturational status or biochemical properties of tumour microvascular endothelium may explain the lack of leucocyte adherence upon PDT.

**Keywords:** photodynamic therapy; Photofrin; leucocyte; endothelium

Selective tumour necrosis occurs upon photodynamic therapy (PDT) through various pathways involving toxic reactive oxygen species. Therapy with the most commonly used photosensitiser Photofrin induces, in particular, damage to tumour microvasculature, cell membranes and mitochondria, resulting in both blood flow stasis and tumour cell death (Henderson and Dougherty, 1992). The early vascular shutdown associated with delayed tumour cell damage suggests that ischaemia-related cell death plays a major role in tumour destruction induced by PDT. An inflammatory reaction with gross oedema and erythema is early observed in illuminated tissue following PDT (Meyer-Betz, 1913; Dougherty *et al.*, 1990). This may be mediated by inflammatory mediators released from mast cells and macrophages (Kerdel *et al.*, 1987), and direct damage to the endothelium, which is highly susceptible to PDT (Gomer *et al.*, 1988; Leunig *et al.*, 1994). Thus an inflammatory response might contribute to the tumour necrosis upon PDT.

The adhesion of neutrophil leucocytes to the microvascular endothelium in response to various noxious stimuli is an initial event in acute inflammatory response and a prerequisite for subsequent leucocyte emigration at sites of inflammation. This interaction between leucocytes and the endothelium is mediated by a repertoire of inducible molecules presented on the membrane of both leucocytes and the endothelial cell (Springer, 1994). Adherent leucocytes, as seen in post-ischaemic reperfusion, contribute to vascular and tissue injury by the release of oxygen-derived free radicals, cytotoxic enzymes, cytokines and inflammatory mediators, and by mechanically reducing vessel diameters or plugging capillaries. The findings of Renard *et al.* (1994) suggest involvement of leucocyte-mediated inflammation as a mechanism in immunotherapy of human tumours. Following treatment with IFN-γ and TNF-α, they found prominent early accumulation of neutrophil leucocytes within the tumour vasculature, preceding lymphocyte and macrophage infiltration and tumour necrosis.

Leucocyte-mediated tissue damage seems to contribute to inflammatory reaction as seen in normal tissue upon PDT, because reduced skin phototoxicity after injection of haematoporphyrin has been observed in leucopenic animals (Lim *et al.*, 1985). In a recent study, Fingar *et al.* (1992) reported that PDT induces leucocyte adherence in microvessels of the rat cremaster muscle. Oxygen radicals as mostly released indirectly by the xanthine oxidase pathway upon PDT (Athar *et al.*, 1989) potentially induce leucocyte adherence (Patel *et al.*, 1991). It seems therefore likely that leucocytes contribute to the tumoricidal mechanisms of PDT. In contrast, recent publications raised evidence that leucocyte-endothelium interaction is diminished in tumours and can only be slightly modulated by mediators inducing the expression of adhesion molecules (Ohkubo *et al.*, 1991; Wu *et al.*, 1992) or by irradiation (Wu *et al.*, 1994). The effect of PDT on leucocyte–endothelium interaction in tumours and their potential role on PDT efficacy have not yet been investigated.

The objective of the present study was therefore to test the hypothesis that PDT might increase leucocyte–endothelium interaction in the microvasculature of tumours. For that purpose we quantified the number of adherent leucocytes, red blood cell velocity and vessel diameters following PDT in the amelanotic melanoma A-Mel-3 of the hamster and in surrounding normal host tissue by intravital microscopy.

### Materials and methods

### Animals and tumour preparation

Experiments were carried out using male Syrian Golden hamsters (6–8 weeks old, 60–80 g body weight) in accordance with institutional guidelines. The animals were housed one per cage and had free access to tap water and standard laboratory food throughout the experiments. A dorsal skinfold chamber preparation consisting of two symmetrical titanium frames was surgically implanted as described earlier in detail (Endrich *et al.*, 1980; Asaishi *et al.*, 1981). Following implantation of the transparent chamber and a recovery period of 48 h from anaesthesia and microsurgery, preparations fulfilling the criteria of an intact microcirculation were utilised for implantation of $2 \times 10^5$ cells of the amelanotic

Correspondence: AE Goetz
*Present address: Department of Radiation Oncology, Massachusetts General Hospital, Boston, MA 02114, USA
Received 30 November 1994; revised 16 June 1995; accepted 21 June 1995

melanoma of the hamster A-Mel-3 (Fortner et al., 1961) into the chamber. PDT and fluorescence microscopy were performed after 5–7 days of tumour growth, when functioning tumour microcirculation was established (mean tumour diameter 4–5 mm). Fine polyethylene catheters (PE10, inner diameter 0.28 mm) were permanently implanted into the right jugular vein and the right carotid artery before photosensitiser injection. All surgical procedures were performed under pentobarbital anaesthesia (50 mg $kg^{-1}$ I.P.; Nembutal; Sanofi-LEVA, Hannover, Germany). The animals tolerated the dorsal skinfold chambers well and showed no signs of discomfort.

### *PDT and experimental groups*

Before injection of the photosensitiser animals were randomly assigned to five groups: controls ($n = 6$), Photofrin only ($n = 6$), laser light only (100 J $cm^{-2}$; $n = 6$), PDT (10 J $cm^{-2}$; $n = 6$), and PDT (100 J $cm^{-2}$; $n = 6$). The photosensitiser Photofrin (5 mg $kg^{-1}$ i.v.; Lederle, Wolfratshausen, Germany) was injected 24 h before PDT. Control animals were injected with 0.3 ml 0.9% saline. For laser irradiation the awake animal was immobilised in a perspex tube and placed on a custom-made stage. An argon pumped dye laser tuned to 630 nm (Aesculap-Meditec, Heroldsberg, Germany) was used to apply light at a power density of 100 mW $cm^{-2}$ via an optical fibre and lens system yielding a uniform beam of 15 mm in diameter. A total light dose of 10 or 100 J $cm^{-2}$ was administered to the whole chamber preparation. Stability of power density and homogeneity of light was controlled with a calibrated power meter (Coherent, Palo Alto, CA, USA).

### *Intravital fluorescence microscopy*

The awake hamster was immobilised and placed on a motorised, computer-controlled $x$–$y$ microscope stage (Kontron GmbH, Eching, Germany) under a modified Leitz microscope (Orthoplan; Leitz, Munich, Germany). Before Photofrin injection, a × 20 long distance objective (Leitz) and transillumination were used to select 5–7 sites of interest per chamber, each containing one or several unbranched post-capillary venules of normal tissue with a minimal distance > 1 mm from the tumour margin, or converging vessels of tumour tissue (15–40 μm diameter, length > 100 μm). The coordinates of the sites of interest were stored on hard disk for subsequent investigation of leucocyte–endothelium interaction, diameter and red blood cell velocity in the identical vessel segments before and at defined times after PDT.

For visualisation by means of intravital fluorescence microscopy, leucocytes were stained *in vivo* by bolus injection of rhodamine 6G (0.3 ml $kg^{-1}$ of a 0.05% solution i.v.; Merck, Darmstadt, Germany). Red blood cell velocity and vessel diameters were visualised after intra-arterial injection of erythrocytes (approximately 1 ml of cells $kg^{-1}$ body weight; labelled with fluorescein isothiocyanate (FITC; Sigma, Deisenhofen, Germany)) according to Zimmerhackl et al. (1983). Selective observation of rhodamine 6G-stained leucocytes was possible using epi-illumination with a 100 W mercury lamp attached to a Ploemopack illuminator with a Leitz N2 filter block (excitation 530–560 nm, emission ≥ 580 nm), and FITC-labelled erythrocytes were visualised using a Leitz I2/3 filter block (excitation 450–490 nm, emission ≥ 515 nm).

Intravital microscopy was performed before, 5, 30, 60 and 180 min after PDT. At each defined time, images were acquired by an SIT video camera (C2400-08; Hamamatsu, Herrsching, Germany) and recorded on video tape (VO-5850; Sony, Munich, Germany). Meticulous care was taken to minimise the light exposure of the tissue. For this purpose, intensity of epi-illumination was controlled by a power meter (Coherent) and reduced to a power density < 1 mW $cm^{-2}$. Exposure of each site of interest to epi-illumination was limited to 4 s (twice 2 s with an intermission of 28 s) for each filter block and each defined time by use of an electromechanical light shutter (Prontor-Magnetic; Hasselblad, Ahrensburg, Germany).

Analysis of microcirculatory parameters was performed from the video tape by means of an image analysis system (Optimas; Bioscan, Edmonds, WA, USA). Sticking leucocytes, i.e. cells adherent to the inner vessel surface, were defined as cells not moving for 30 s and are given as the number of leucocytes per $mm^2$ of vessel wall calculated from inner vessel diameter and length (100–150 μm) of the vessel segment studied. Red blood cell velocity was measured as the distance in axial direction up to ten centre-flowing erythrocytes passed per time. Wall shear rate ($\gamma$) in vessels of tumour and normal tissue was calculated based on the Newtonian definition: $\gamma = 8 \times V \times D^{-1}$ where $V$ represents the mean red blood cell velocity ( = centre-line velocity × 1.6; Lipowsky and Zweifach, 1978) and $D$ the diameter of the individual microvessel.

### *Statistical analysis*

All results are given as means ± s.e.m. Non-parametric one-way analysis of variance and multiple comparison on ranks of several independent samples were performed using the Kruskal–Wallis test (Theodorsson-Norheim, 1986). $P$-values smaller than 5% were regarded as significant.

### Results

### *Leucocyte–endothelium interaction*

Already under baseline conditions, a markedly lower number of adherent leucocytes ($P < 0.01$) was observed in tumour microvessels (7 ± 1) as compared with post-capillary and collecting venules of normal tissue (31 ± 9 leucocytes $mm^{-2}$, mean ± s.e.m.). Images of leucocytes adhering to microvessels in normal and tumour tissue are shown in Figure 1. In tumour microvessels, the number of adherent leucocytes did not change after PDT (Figure 2). In contrast, an approximately 3-fold increase in the number of adhering leucocytes ($P < 0.01$) was observed in post-capillary venules of normal tissue (Figure 3). Similar values of adherent leucocytes were documented after PDT with 10 and 100 J $cm^{-2}$, yielding a maximum 1 h following treatment to 286% and 366% of baseline values respectively ($P < 0.01$). Treatment with Photofrin alone or laser light alone exhibited no effects on leucocyte adherence in comparison with controls.

### *Red blood cell velocity*

In control groups and before treatment, blood flow velocity in tumour vessels was below the values in venules of surrounding normal tissue ($P < 0.05$, Figures 4 and 5). PDT of the A-Mel-3 tumour induced a rapid decrease of red blood cell velocity (Figure 4), until 3 h following PDT with 10 J $cm^{-2}$, tumour blood flow velocity decreased to 30% of the corresponding saline-treated controls ($P < 0.05$). A rapid standstill of red blood cells in most of the tumour microvessels was observed after PDT with 100 J $cm^{-2}$, lasting throughout the entire observation period ($P < 0.01$). In contrast, PDT revealed no changes in blood cell velocity in post-capillary venules and collecting venules of normal tissue (Figure 5).

### *Vessel diameters and wall shear rate*

As shown in Table I, diameters of the tumour microvessels studied were below diameters of post-capillary and collecting venules of normal tissue ($P < 0.01$). Treatment did not provoke any changes in vessel diameters. Rolling and adhesion of leucocytes is partly influenced by the wall shear rate. Therefore, this parameter was calculated from diameters of individual vessels and red blood cell velocity, and is depicted in Figures 6 and 7. Under baseline conditions, wall shear rate was similar in tumour (73.9 ± 16.2 $s^{-1}$) and normal tissue

**Figure 1** Leucocyte adhesion before (**a**,**b**) and 1 h after PDT (**c**,**d**; 10 J $cm^{-2}$) in a post-capillary venule of normal tissue (**a**,**c**) and in a tumour microvessel (**b**,**d**). Leucocytes are visualised as white dots by *in vivo* staining with rhodamine 6G. Bar represents 0.1 mm.

**Figure 2** Number of adherent leucocytes per $mm^2$ of endothelial surface in convergent microvessels of the A-Mel-3 tumour before and 5 min, 30 min, 1 h and 3 h after therapy in the following groups: saline-treated controls ( , $n = 6$), Photofrin alone ( , $n = 6$), laser light alone (100 J $cm^{-2}$, , $n = 6$), PDT with 10 J $cm^{-2}$ ( , $n = 6$), PDT with 100 J $cm^{-2}$ ( , $n = 6$). Because red blood cell velocity rapidly decreased to a standstill in the group treated with 100 J $cm^{-2}$ PDT, the number of adhering leucocytes after treatment could not be quantified in this group. Values are means ± s.e.m.

**Figure 4** Red blood cell velocity (mm $s^{-1}$) in convergent microvessels of the A-Mel-3 tumour before and 5 min, 30 min, 1 h and 3 h after therapy. Bars represent the following groups: controls ( , $n = 6$), Photofrin alone ( , $n = 6$), laser light alone (100 J $cm^{-2}$, , $n = 6$), PDT with 10 J $cm^{-2}$ ( , $n = 6$), PDT with 100 J $cm^{-2}$ ( , $n = 6$). Following PDT with 100 J $cm^{-2}$, red blood cell velocity decreased to a standstill. Means ± s.e.m.; $^*P < 0.05$, $^{**}P < 0.01$ *vs* saline-treated controls.

**Figure 3** Number of adherent leucocytes per $mm^2$ of endothelial surface in post-capillary and collecting venules of striated muscle before and 5 min, 30 min, 1 h and 3 h after therapy with Photofrin alone ( , $n = 6$), laser light alone (100 J $cm^{-2}$, , $n = 6$), PDT with 10 J $cm^{-2}$ ( , $n = 6$), PDT with 100 J $cm^{-2}$ ( , $n = 6$), as well as in saline-treated controls ( , $n = 6$). Means ± s.e.m.; $^*P < 0.05$, $^{**}P < 0.01$ *vs* saline-treated controls.

**Figure 5** Red blood cell velocity (mm $s^{-1}$) in post-capillary and collecting venules of striated muscle before and 5 min, 30 min, 1 h and 3 h after therapy in the following groups: Photofrin alone ( , $n = 6$), laser light alone (100 J $cm^{-2}$, , $n = 6$), PDT with 10 J $cm^{-2}$ ( , $n = 6$), PDT with 100 J $cm^{-2}$ ( , $n = 6$), as well as saline-treated controls ( , $n = 6$). Means ± s.e.m.

**Table I** Vessel diameter (μm) in tumour and normal tissue

| *Region* | *Group* | *Before* | *Time after treatment* 5 *min* | 30 *min* | 1 *h* | 3 *h* |
|---|---|---|---|---|---|---|
| Tumour | Control | 16.9 ± 1.4 | 17.5 ± 1.2 | 17.3 ± 1.3 | 17.1 ± 1.2 | 17.7 ± 1.7 |
| | Photofrin only | 19.8 ± 2.4 | 19.8 ± 1.1 | 20.0 ± 1.2 | 20.4 ± 1.9 | 21.8 ± 2.4 |
| | Laser light only | 17.0 ± 1.2 | 17.1 ± 1.6 | 16.6 ± 1.0 | 16.5 ± 0.9 | 16.3 ± 1.3 |
| | PDT, 10 J $cm^{-2}$ | 23.4 ± 2.1 | 25.6 ± 2.5 | 23.0 ± 2.5 | 23.8 ± 1.5 | 16.8 ± 5.6 |
| | PDT, 100 J $cm^{-2}$ | 22.0 ± 4.3 | 23.3 ± 2.8 | 22.5 ± 1.5 | 22.5 ± 1.9 | 20.6 ± 1.9 |
| Normal tissue | Control | 30.6 ± 2.2 | 30.2 ± 2.5 | 30.7 ± 1.5 | 29.8 ± 1.9 | 32.1 ± 1.9 |
| | Photofrin only | 30.3 ± 1.9 | 30.4 ± 2.5 | 30.7 ± 2.1 | 32.3 ± 1.4 | 29.6 ± 3.4 |
| | Laser light only | 33.8 ± 6.4 | 35.0 ± 5.6 | 33.1 ± 6.1 | 31.0 ± 2.1 | 31.9 ± 3.5 |
| | PDT, 10 J $cm^{-2}$ | 32.2 ± 2.9 | 30.8 ± 4.0 | 32.2 ± 2.7 | 34.7 ± 1.3 | 31.0 ± 1.6 |
| | PDT, 100 J $cm^{-2}$ | 33.7 ± 4.4 | 33.3 ± 6.5 | 31.6 ± 2.0 | 33.2 ± 2.7 | 31.5 ± 2.1 |

All data are given as means ± s.e.m.

**Figure 6** Wall shear rate ($s^{-1}$) in convergent microvessels of the A-Mel-3 tumour before and 5 min, 30 min, 1 h and 3 h after therapy. Bars represent the following groups: Saline-treated controls (□, *n* = 6), Photofrin alone (▨, *n* = 6), laser light alone (100 J $cm^{-2}$, ▧, *n* = 6), PDT with 10 J $cm^{-2}$ (▦, *n* = 6), PDT with 100 J $cm^{-2}$ (■, *n* = 6). Following PDT with 100 J $cm^{-2}$, shear rate was reduced to zero in association with the standstill of blood flow velocity. Means ± s.e.m.; $*P<0.05$, $**P<0.01$ *vs* saline-treated controls.

**Figure 7** Venular wall shear rate ($s^{-1}$) in striated muscle before and 5 min, 30 min, 1 h and 3 h after therapy in the following groups: Photofrin alone (▨, *n* = 6), laser light alone (100 J $cm^{-2}$, ▧, *n* = 6), PDT with 10 J $cm^{-2}$ (▦, *n* = 6), PDT with 100 J $cm^{-2}$ (■, *n* = 6), as well as controls (□, *n* = 6). Means ± s.e.m.

microvessels (64.5 ± 11.6 $s^{-1}$). Following PDT with 10 J $cm^{-2}$, the shear rate decreased significantly in tumour microvessels ($P<0.05$) in association with the decrease in blood flow velocity (Figure 6). No changes of wall shear rate were noticed in venules of normal tissue following PDT (Figure 7).

### Discussion

The present study was based on the hypothesis that PDT might induce leucocyte adhesion in microvessels of normal and tumour tissue. Subsequently, the adhesion of leucocytes could potentially contribute to the observed microvascular damage. Also, reduction of the vessel lumen by leucocytes adhering to the endothelium could increase microvascular resistance to flow and contribute to the microvascular shutdown observed after PDT. The data presented, however, demonstrate clearly that PDT induced leucocyte adhesion in microvessels of normal tissue, but not in tumour tissue.

### *Methods*

PDT exerts its toxicity upon the activation of a photosensitiser by light in the presence of oxygen. The observation of leucocyte–endothelium interaction requires light and could potentially interfere with PDT by increasing the light dose. For that reason we took special precautions to limit and standardise the light exposure to the tissue and included control groups to determine the effects of fluorescence microscopy itself. To reduce the time of epi-illumination for observation, we restricted the analysis on adherence of leucocytes, red blood cell velocity and vessel diameters. Evaluation of rolling leucocytes was omitted to avoid the 8-fold higher light exposure necessary for this measurement. Rolling of leucocytes regularly precedes their adherence, to generate their destructive potential tight adherence is necessary (Springer, 1994). The results obtained in the control group treated with Photofrin prove that fluorescence microscopy did not affect our measured parameters.

### *Effects on normal tissue*

In normal tissue, rolling and adhesion of leucocytes are almost exclusively found in post-capillary venules and collecting venules (Atherton and Born, 1972; Nolte *et al*., 1991) which are the sites of leucocyte emigration, whereas little or no such activity is observed in arterioles (Ley and Gaehtgens, 1991). Our results obtained in normal microcirculation are in agreement with findings from a study by Fingar *et al*. (1992) demonstrating a marked increase in leucocyte adhesion to venules of the rat cremaster muscle following PDT. This study also suggested that leucocyte adhesion is not involved in the observed increase of vascular permeability after PDT, because pretreatment with indomethacin inhibited albumin extravasation, but did not change the number of adherent leucocytes. These results may be specific for the normal rat cremaster muscle preparation exposed to high doses of Photofrin (10–25 mg $kg^{-1}$), not used clinically.

The first signs of response to PDT are oedema and erythema formation whenever skin is contained in the treatment field (Henderson and Dougherty, 1992). A release of inflammatory and immune mediators has been observed following PDT, namely eicosanoids from tumour cells and vascular endothelium (Henderson and Donovan, 1989), and histamine (Kerdel *et al*., 1987) and tumour necrosis factor (Evans *et al*., 1990) from mast cells (Kamide *et al*., 1984). In addition, PDT-induced microvascular damage can be partially inhibited by prostanoid antagonists (Reed *et al.*, 1991; Fingar *et al.*, 1993). An immune response is also suggested by the observed infiltration of PDT-treated tissue with lymphocytes, plasma cells and histiocytes (Shumaker and Hetzel, 1987).

The interaction between leucocytes and endothelial cells follows a multistep process mediated by specific adhesion receptor molecules (Springer, 1994). The adhesion of leucocytes to the vascular endothelium requires their expression. Activation of endothelial cells and/or leucocytes directly by PDT or by local accumulation of inflammatory mediators such as histamine may induce the presentation of adhesion receptors. Reactive oxygen species occurring during the reperfusion period after organ ischaemia have been demonstrated to contribute to leucocyte adherence (Nolte *et al.*, 1991). Oxygen radicals, which are also released by PDT, induce the expression of GMP-140 on the surface of endothelial cells, a membrane glycoprotein mediating rolling of leucocytes (Patel *et al.*, 1991). GMP-140, also known as P-selectin, is stored preformed in the Weibel–Palade bodies of endothelial cells and rapidly mobilised to the plasma membrane upon activation. Rolling of leucocytes is considered to be a prerequisite for leucocyte adherence (Springer, 1994), thus P-selectin may especially be involved in the early neutrophil adhesion observed following PDT. The time delay between PDT and adhesion of leucocytes suggests that induced expression of adhesion receptors following *de novo* synthesis, e.g. E-selectin and intercellular adhesion molecule-1 (ICAM-1), has contributed to the increased number of adherent leucocytes 1 and 3 h after PDT. Because red blood cell velocity, vessel diameters and wall shear rate did not change following PDT in normal tissue, a reduction in the shear rate cannot have accounted for the increase in the number of adherent leucocytes in normal tissue.

The severe inflammatory response regularly observed in PDT-treated skin was accompanied by a marked increase in the number of adhering leucocytes. Light exposure of normal tissues surrounding the tumour has been demonstrated to enhance the tumoricidal effect of PDT by destruction of the 'tumour bed' (Fingar and Henderson, 1987). Activation and adherence of leucocytes to the microvascular endothelium is a prerequisite for their migration across the vascular wall (von Andrian *et al.*, 1991). The induction of leucocyte adherence in normal tissue suggests that leucocyte-mediated tissue damage potentially contributes to destruction of tumour bed. In addition, the emigration of activated leucocytes from the tumour margin could possibly further contribute to the tumoricidal effect of PDT.

*Effects on tumour tissue*

The present study demonstrates that PDT does not induce leucocyte adhesion in tumour tissue. Owing to the rapid decline of tumour blood flow after PDT with 100 J $cm^{-2}$, leucocyte adhesion could not be quantified in this group. PDT of the A-Mel-3 tumour with 100 J $cm^{-2}$ results in complete tumour remission (Leunig, M *et al.*, 1994). Because red blood cell velocity decreased rapidly to a standstill in this group, we investigated a second group of animals treated with a subtherapeutic light dose of 10 J $cm^{-2}$, which resulted in flow retardation and allowed a prolonged observation of leucocyte–endothelium interaction. Increased margination of leucocytes and their primary interaction with the endothelium may be due to reduction of red blood cell velocity and lowered shear stress. The experiments demonstrated a significant reduction of wall shear stress in tumour vessels following PDT. However, our intravital microscopic observations revealed that even the leucocytes entrapped because of stasis in tumour microvessels following the higher light dose did not interact with the endothelium. The reduction of red blood cell velocity in tumour microvessels following PDT, whereas venule and tumour vessel diameters remained unchanged, is in accordance with results from earlier studies (Goetz *et al.*, 1987; Reed *et al.*, 1989).

The effects of radiation therapy on leucocyte–endothelium interaction in tumour and normal tissue have recently been studied by Wu *et al.* (1994). These authors have shown that irradiation does not change the number of adherent leucocytes in centre vessels of the tumour, whereas it significantly increases in normal tissue preparations. In contrast, leucocyte adherence is reduced following radiation in vessels at the tumour periphery and in vessels of adjacent tissue. This observation may in part be explained by the possibility that vessels at the tumour periphery may have been vessels originating from normal tissue which have been invaded by the tumour. Vessels in the tumour centre may have had their origin from tumour neoangiogenesis and therefore exhibited different endothelial properties. Preliminary observations on venules of normal tissue *close to the tumour margin* also indicate a reduced adhesion of leucocytes following PDT (data not shown). These findings may be explained by the release of substances from the tumour parenchyma which inhibit leucocyte adherence.

Our results confirm observations of a diminished leucocyte–endothelium interaction in tumour tissue, as reported by Wu *et al.* (1992, 1994) for a rat mammary adenocarcinoma. The finding that leucocytes did not interact with the tumour endothelium even at significantly reduced wall shear stresses suggests that adhesion receptors were not, or not adequately, expressed. Indeed, by immunohistochemistry a reduced expression of leucocyte adhesion molecules in human vascular tumours has been observed under baseline conditions in comparison with various normal tissues (Kuzu *et al.*, 1993). In contrast, Renard *et al.* (1994) have found an increased expression of E-selectin on the endothelium of human melanomas and sarcomas after regional treatment with IFN-$\gamma$ and TNF-$\alpha$, which was associated with neutrophil accumulation and inflammation in tumour tissue. These observations suggest a remarkable inter-tumour variability of the properties of tumour endothelium. In addition, the stimuli required for the expression of adhesion molecules may differ between the endothelium of normal tissue and tumours.

In conclusion, the current study has shown that tumour destruction induced by PDT is not mediated by leucocyte–endothelium interaction in the tumour. In normal tissue, the observed increase in the number of adherent leucocytes may contribute to the inflammatory reaction. The different reactivity to the stimulation by PDT may be explained by a different maturational state of the neoplastic microvascular endothelial cells.

**Acknowledgements**

The authors would like to thank Professor Dr Dr hc K Messmer for his critical comments on the manuscript. This investigation was supported by a grant of the Bundesministerium für Forschung und Technologie to AE Goetz (Grant No. 0706903A5). M Dellian is a recipient of a Feodor-Lynen Fellowship from the Alexander von Humboldt Foundation.

### References

ASAISHI K, ENDRICH B, GOETZ A AND MESSMER K. (1981). Quantitative analysis of microvascular structure and function in the amelanotic melanoma A-Mel-3. *Cancer Res.*, **41**, 1898–1904

ATHAR M, ELMETS CA, BICKERS DR AND MUKHTAR H. (1989). A novel mechanism for the generation of superoxide anions in hematoporphyrin derivative-mediated cutaneous photosensitization. *J. Clin. Invest.*, **83**, 1137–1143.

ATHERTON A AND BORN GVR. (1972). Quantitative investigations of the adhesiveness of circulating polymorphonuclear leukocytes to blood vessel walls. *J. Physiol.*, **222**, 447–474.

DOUGHERTY TJ, COOPER MT AND MANG TS. (1990). Cutaneous phototoxic occurences in patients receiving Photofrin. *Lasers Surg. Med.*, **10**, 485–488.

ENDRICH B, ASAISHI K, GOETZ A AND MESSMER K. (1980). Technical report. A new chamber technique for microvascular studies in unanesthetized hamsters. *Res. Exp. Med. Berl.*, **177**, 125-134.
EVANS S, MATTHEWS W, PERRY R, FRAKER D, NORTON J AND PASS HI. (1990). Effect of photodynamic therapy on tumor necrosis factor production by murine macrophages. *J. Natl Cancer Inst.*, **82**, 34-39.
FINGAR VH AND HENDERSON BW. (1987). Drug and light dose dependence of photodynamic therapy: a study of tumor and normal tissue response. *Photochem. Photobiol.*, **46**, 837-841.
FINGAR VH, WIEMAN TJ, WIEHLE SA AND CERRITO PB. (1992). The role of microvascular damage in photodynamic therapy: the effect of treatment on vessel constriction, permeability, and leukocyte adhesion. *Cancer Res.*, **52**, 4914-4921.
FINGAR VH, SIEGEL KA, WIEMAN TJ AND DOAK KW. (1993). The effects of thromboxane inhibitors on the microvascular and tumor response to photodynamic therapy. *Photochem. Photobiol.*, **58**, 393-399.
FORTNER JG, MAHY AG AND SCHRODT GR. (1961). Transplantable tumors of the Syrian (Golden) hamster. Part I: tumors of the alimentary tract, endocrine glands and melanomas. *Cancer Res.*, **21**, 161-198.
GOETZ AE, KÖNIGSBERGER R, FEYH J, CONZEN PF AND LUMPER W. (1987). Breakdown of tumor microcirculation induced by shock-waves or photodynamic therapy. In *Surgical Research: Recent Concepts and Results*, Messmer K and Baethmann A. (eds). pp. 81-93. Springer: Berlin.
GOMER CJ, RUCKER N AND MURPHREE AL. (1988). Differential cell photosensitivity following porphyrin photodynamic therapy. *Cancer Res.*, **48**, 4539-4542.
HENDERSON BW AND DONOVAN JM. (1989). Release of prostaglandin $E_2$ from cells by photodynamic treatment in vitro. *Cancer Res.*, **49**, 6896-6900.
HENDERSON BW AND DOUGHERTY TJ. (1992). How does photodynamic therapy work? *Photochem. Photobiol.*, **55**, 145-157.
KAMIDE R, GIGLI I AND LIM HW. (1984). Participation of mast cells and complement in the immediate phase of hematoporphyrin-induced phototoxicity. *J. Invest. Dermatol.*, **82**, 485-490.
KERDEL FA, SOTER NA AND LIM HW. (1987). In vivo mediator release and degranulation of mast cells in hematoporphyrin derivative-induced phototoxicity in mice. *J. Invest. Dermatol.*, **88**, 277-280.
KUZI I, BICKNELL R, FLETCHER CDM AND GATTER KC. (1993). Expression of adhesion molecules on the endothelium of normal tissue vessels and vascular tumors. *Lab. Invest.*, **69**, 322-328.
LEUNIG A, STAUB F, PETERS J, HEIMANN A, CSAPO C, KEMPSKI O AND GOETZ AE. (1994). Relation of early photofrin uptake to photodynamically induced phototoxicity and changes in cell volume in different cell lines. *Eur. J. Cancer*, **30A**, 78-83.
LEUNIG M, LEUNIG A, LANKES P AND GOETZ AE. (1994). Evaluation of photodynamic therapy-induced heating of hamster melanoma and its effect on local tumor eradication. *Int. J. Hyperthermia*, **10**, 297-306.
LEY K AND GAEHTGENS P. (1991). Endothelial, not hemodynamic, differences are responsible for preferential leukocyte rolling in rat mesenteric venules. *Circ. Res.*, **69**, 1034-1041.
LIM HW, YOUNG L, HAGAN M AND GIGLI I. (1985). Delayed phase of hematoporphyrin-induced phototoxicity: modulation by complement, leukocytes, and antihistamines. *J. Invest. Dermatol.*, **84**, 114-117.
LIPOWSKY H AND ZWEIFACH BW. (1978). Technical report. Application of the 'two slit' photometric technique to the measurement of microvascular volumetric flow rates. *Microvasc. Res.*, **15**, 93-101.
MEYER-BETZ F. (1913). Untersuchungen über die biologische (photodynamische) Wirkung des Hämatoporphyrins und anderer Derivate des Blut- und Gallenfarbstoffes. *Dtsch. Arch. Klin. Med.*, **112**, 476-481.
NOLTE D, LEHR HA AND MESSMER K. (1991). Adenosine inhibits postischemic leukocyte-endothelium interaction in postcapillary venules of the hamster. *Am. J. Physiol.*, **261**, H651-H655.
OHKUBO C, BIGOS D AND JAIN RK. (1991). Interleukin 2 induced leukocyte adhesion to the normal and tumor microvascular endothelium in vivo and its inhibition by dextran sulfate: implications for vascular leak syndrome. *Cancer Res.*, **51**, 1561-1563.
PATEL KD, ZIMMERMANN GA, PRESCOTT SM, MCEVER RP AND MCINTYRE TM. (1991). Oxygen radicals induce human endothelial cells to express GMP-140 and bind neutrophils. *J. Cell Biol.*, **112**, 749-760.
REED MW, WIEMAN TJ, SCHUSCHKE DA, TSENG MT AND MILLER FN. (1989). A comparison of the effects of photodynamic therapy on normal and tumor blood vessels in the rat microcirculation. *Radiat. Res.*, **119**, 542-552.
REED MW, SCHUSCHKE DA AND MILLER FN. (1991). Prostanoid antagonists inhibit the response of the microcirculation to 'early' photodynamic therapy. *Radiat. Res.*, **127**, 292-296.
RENARD N, LIENARD D, LESPAGNARD L, EGGERMONT A, HEIMANN R AND LEJEUNE F. (1994). Early endothelium activation and polymorphonuclear cell invasion precede specific necrosis of human melanoma and sarcoma treated by intravascular high-dose tumour necrosis factor alpha (rTNFa). *Int. J. Cancer*, **57**, 656-663.
SHUMAKER BP AND HETZEL FW. (1987). Clinical laser photodynamic therapy in the treatment of bladder cancer. *Photo chem. Photobiol.*, **46**, 899-901.
SPRINGER TA. (1994). Traffic signals for lymphocyte recirculation and leukocyte emigration: the multistep paradigm. *Cell*, **76**, 301-314.
THEODORSSON-NORHEIM E. (1986). Kruskal-Wallis test: BASIC computer program to perform nonparametric one-way analysis of variance and multiple comparisons on ranks of several independent samples. *Computer Methods and Programs in Biomedicine*, **23**, 57-62.
VON ANDRIAN UH, CHAMBERS JD, MCEVOY LM, BARGATZE RF, ARFORS KE AND BUTCHER EC. (1991). Two-step model of leukocyte-endothelial cell interaction in inflammation: distinct roles for LECAM-1 and the leukocyte $b_2$ integrins in vivo. *Proc. Natl Acad. Sci. USA*, **88**, 7538-7542.
WU NZ, KLITZMAN B, DODGE R AND DEWHIRST MW. (1992). Diminished leukocyte-endothelium interaction in tumor microvessels. *Cancer Res.*, **52**, 4265-4268.
WU NZ, ROSS BA, GULLEDGE C, KLITZMAN B, DODGE R AND DEWHIRST MW. (1994). Differences in leucocyte-endothelium interactions between normal and adenocarcinoma bearing tissues in response to radiation. *Br. J. Cancer*, **69**, 883-889.
ZIMMERHACKL B, PAREKH M, BRINKHUS H AND STEINHAUSEN M. (1983). The use of fluorescent labelled erythrocytes for intravital investigation of flow and local hematocrit in glomerular capillaries in the rat. *Int. J. Microcirc. Clin. Exp.*, **2**, 119-130.